# Mediation effect of TyG-BMI, LDL-C, and CRP on physical activity-stroke risk relationship

**DOI:** 10.1038/s41598-026-63670-1

**Published:** 2026-07-23

**Authors:** Minmin Xu, Qiongyan Tang, Xuemei Xiao, Xinhang Wen, Quanjun Lyu, Weimin Yang

**Affiliations:** 1 Department of Nutrition and Food Hygiene, College of Public Health, Zhengzhou University, Zhengzhou, 450000 China; 2 Department of Neurology, The First Affiliated Hospital, Zhengzhou University, Zhengzhou, 450000 China; 3 Zhengzhou Health College, Zhengzhou, 450000 China

**Keywords:** Physical activity, Stroke, TyG-BMI, LDL-C, CRP, Mediation analysis, Cardiology, Diseases, Endocrinology, Health care, Medical research, Risk factors

## Abstract

While physical activity (PA) is known to reduce stroke risk, the unified biological mechanisms involving metabolic, lipid, and inflammatory pathways remain unclear. This study quantified the mediating roles of TyG-BMI, LDL-C, and CRP in the PA-stroke association using nationwide longitudinal data, and explored subgroup differences. Data from 6269 participants in the China Health and Retirement Longitudinal Study were analyzed. PA was assessed via IPAQ. Triglycerides, blood glucose, CRP, and LDL-C were measured enzymatically; TyG-BMI was calculated as log[triglycerides × fasting glucose/2] × BMI. Chi-square tests, generalized structural equation modeling (GSEM), and interaction tests were used for analyses. A total of 307 incident strokes occurred. In the crude comprehensive mediation model, the PA-stroke association was mediated by TyG-BMI (7.00%), LDL-C (2.21%), and CRP (1.32%). After full adjustment, only the TyG-BMI pathway remained statistically significant, accounting for 10.64% of the total association (OR 0.985, 95% CI 0.976–0.995), whereas the LDL-C and CRP pathways were attenuated and no longer significant. The direct effect accounted for 89.36% of the association in the fully adjusted comprehensive model. Subgroup analysis revealed more pronounced TyG-BMI mediation in adults ≥ 60 years and hypertensive patients, but not in diabetic patients. Greater PA was associated with a lower risk of incident stroke, partly mediated by TyG-BMI. LDL-C and CRP did not show significant mediation after multivariable adjustment. Personalized PA interventions for those at high metabolic risk are crucial for stroke prevention. Future research should elucidate the uncharacterized direct-effect pathways.

**Supplementary Information:**

The online version contains supplementary material available at 10.1038/s41598-026-63670-1.

## Introduction

Stroke remains a major global health challenge and one of the leading cause of death and long-term disability. Its substantial socioeconomic burden highlights the need for effective primary prevention strategies^1,2^. Among modifiable risk factors, physical inactivity is consistently associated with increased stroke risk. Large prospective cohorts, including studies from Canada and the UK Biobank, have associated regular moderate-to-vigorous physical activity (PA) with lower incidence and mortality of both ischemic and hemorrhagic stroke^3,4^. Accordingly, promoting of PA is a cornerstone of international guidelines for the prevention of cardiovascular and cerebrovascular diseases^5^.

The biological pathways linking PA to stroke risk remain incompletely understood. PA may influence stroke risk through multiple intermediate physiological processes rather than a single pathway^6^. Insulin resistance^7^, dyslipidemia^8^, and chronic inflammation^9^ are important processes in stroke pathogenesis. The triglyceride-glucose body mass index (TyG-BMI) , which combines the triglyceride-glucose index with body mass index, is a practical marker of insulin resistance and metabolic dysfunction^10,11^. Higher TyG-BMI has been associated with increased stroke risk, particularly ischemic stroke^12,13^. Low-density lipoprotein cholesterol (LDL-C) is a causal factor in atherosclerosis^11^ and a major modifiable risk factors for atherosclerotic stroke^14,15^. C-reactive protein (CRP), a marker of systemic inflammation, has also been associated with vascular injury^16^ and stroke risk. PA has been associated with improve insulin sensitivity and weight control^17^, lower LDL-C^18^, and reduced chronic inflammation^19^ . Together, these observations provide a rationale for examining TyG-BMI, LDL-C, and CRP as potential intermediate pathways linking PA to stroke risk.

These observations identify TyG-BMI, LDL-C, and CRP as plausible candidate mediators of the association between PA and stroke. In observational studies, mediation analysis can quantify the extent to which this association is statistically consistent with indirect pathways throughcandidate biomarkers^20^. Previous studies, including Mendelian randomization analyses, suggest that body mass index may partly mediates the association between PA and stroke^21^. However, most studies have considered these mediators separately rather than evaluating their interrelated roles within a unified analytical framework. Simultaneous assessments of TyG-BMI, LDL-C, and CRP as parallel mediators therefore remain scarce. This question is particularly relevant in China^22^, where PA patterns among middle-aged and older adults may differ from those in Western populations.

Using data from a large national longitudinal cohort, we examined the association between PA and incident stroke and assessed the potential parallel mediating roles of TyG-BMI, LDL-C, and CRP. We hypothesize that higher PA would be associated with a lower risk of incident stroke, partly through favorable profiles of these biomarkers. We further hypothesized that TyG-BMI would show a stronger estimated mediating contribution than LDL-C or CRP, and that the estimated mediation patterns would vary across demographic and clinical subgroups. By comparing the relative contributions of these pathways, we sought to clarify the potential biological pathways underlying the association between PA and stroke.

## Methods

### Study design and participants

The China Health and Retirement Longitudinal Study(CHARLS) is a nationally representative longitudinal survey among the middle-aged and older aged 45 years and above in China. Using a stratified, multi-stage probability sampling design, CHARLS recruited participants from 150 county-level units across 28 provinces. Since its baseline survey in 2011, trained interviewers have conducted face-to-face computer-assisted interviews with participants every two years.

This study used participant-level data from the 2011 baseline survey and the 2015 follow-up wave, linked by unique participant identifiers, covering a four-year observation period. Participants with missing information on demographic characteristics, health status, physical activity assessment, or blood biomarkers were excluded from the analysis. All data used in this study were obtained from the publicly available CHARLS database. The CHARLS study was approved by the Biomedical Ethics Review Committee of Peking University (IRB00001052-11,014), and all participants provided written informed consent before data collection.

### Physical activity(PA)

Participants’ physical activity (PA) levels were measured using the International Physical Activity Questionnaire (IPAQ)^23^. The measurement captured activities lasting at least 10 min per session and classified them as Vigorous Physical Activity (VPA), Moderate Physical Activity (MPA), Light Physical Activity (LPA). VPA included weight-bearing exercise, digging, ploughing, aerobic exercise, fast cycling, and cycling while carrying loads. MPA included carrying light loads, cycling at a usual pace, mopping, tai chi, and brisk walking. LPA included walking at work or at home, as well as walking for recreation, exercise, or leisure. For each reported activity type, participants provided its weekly frequency (1–7 days) and usual daily duration. Daily duration was recorded in four categories: ≥ 10 to < 30 min, ≥ 30 min to < 2 h, ≥ 2 to < 4 h, and ≥ 4 h. These data were used to characterize the frequency and duration of PA.

As CHARLS IPAQ lacks exact continuous PA minutes and only provides categorized duration ranges, we assigned the midpoint of each bracket to generate continuous duration values, consistent with methods used in previous CHARLS-based studies^24^. Specifically: The segment of " ≥ 10 min and < 30 min" was recorded as 20 min; " ≥ 30 min and < 2 h" was recorded as 75 min (converted from 1.25 h); " ≥ 2 h and < 4 h" was recorded as 180 min (converted from 3 h); " ≥ 4 h" was recorded as 240 min (converted from 4 h). The weekly duration score for each PA intensity level was calculated as the product of the weekly frequency (days/week) and the daily time spent (min/day) for that specific level. The total physical activity volume (PAV) score was expressed in metabolic equivalents (METs)^25^, using the following formula: PAV = 8.0 × Weekly VPA Duration Score + 4.0 × Weekly MPA Duration Score + 3.3 × Weekly LPA Duration Score.

### Assessment of TyG-BMI, CRP, LDL-C

Venous blood samples were transported from local Center(s) for Disease Control and Prevention(CDC) in Beijing within two weeks of collection. Upon receipt, these samples were stored at −20 °C. Following laboratory processing, the samples were transferred to a −80 °C freezer for long-term storage. Serum triglycerides, fasting blood glucose, ReactiveProtein(CRP) and Low-Density Lipoprotein Cholesterol(LDL-C) concentrations were measured using enzyme colorimetric assay at [verify: the Chinese Academy of Medical Sciences or the clinical laboratory of You’an Hospital, Capital Medical University]. TyG-BMI were calculated as ln [triglycerides (mg/dL) × fasting blood glucose (mg/dL)/2] × BMI^26^.

### Incident stroke

Incident stroke was defined as a new self-reported physician diagnosis of stroke during follow-up among participants without stroke at baseline. In CHARLS, participants were asked whether a doctor had ever diagnosed them with stroke. Participants reporting no stroke at baseline but reporting physician-diagnosed stroke during follow-up were classified as incident stroke cases.

### Assessment of other variables

Information on participants’ demographic characteristics, lifestyle factors, and health status was collected using a structured questionnaire. Demographic characteristics included Gender, age, residential area, and marital status; Lifestyle factors included smoking status, alcohol consumption, educational level, and mean daily sleep duration. Health-related indicators included obesity status and disease status. All questionnaire items included response options to promote standardized and consistent data collection.

### Statistical analysis

All analyses were conducted using Stata 18 and RStudio. A *P* value < 0.05 was considered statistically significant. Categorical variables were summarized as n (%) and compared using the chi-square test. Non-normally distributed continuous variables were summarized as the median (interquartile range, IQR) and compared using the Wilcoxon rank-sum test.

Generalized structural equation modeling (GSEM) was used to assess the potential mediating roles of LDL-C, TyG-BMI and CRP in the association between changes in physical activity and incident stroke. Within the GSEM framework, estimated total effects were decomposed into direct and indirect effects. The proportions mediated was calculated as (specific effect/total effect) × 100%, path coefficients were reported as β coefficients, and associations were reported as odds ratios (OR) and 95% confidence intervals(CIs).

Subgroup analyses were conducted by age (≥ 60 versus < 60 years), hypertension status and diabetes status. Interaction *P*-values were calculated for PA-by-subgroup terms to assess heterogeneity in the PA-stroke association. All adjusted analyses controlled for prespecified potential confounders.

Before fitting the mediation models, we constructed an a priori directed acyclic graph (DAG) based on previous literature and clinical knowledge to identify variables that could confound the association between PA and stroke and the mediator-outcome associations. The DAG included demographic factors, lifestyle factors, comorbidities, sleep duration, residential area, and renal disease. Variables identified as potential confounders were included in the fully adjusted model, whereas the candidate mediators (TyG-BMI, LDL-C, and CRP) were modeled as parallel mediating pathways rather than as adjustment covariates for the total PA-stroke association.

## Results

### Characteristics of the participants

This analysis included 6269 participants, of whom 307 developed incident stroke during follow-up, corresponding to an incidence rate of 4.90% (Table 1). Compared with participants who remained stroke-free, those who developed stroke were older and more likely to live in urban areas. They were less likely to be married and less likely to report adequate sleep duration of 7–8 h per day. Physical activity levels were similar between the two groups at baseline in 2011, but participants who developed stroke had lower activity levels in 2015 and a smaller increase in physical activity over follow-up. Hypertension, diabetes, dyslipidemia, and renal disease were more common among participants who developed stroke. In addition, this group had higher levels of LDL-C, CRP, BMI, and TyG-BMI, indicating a greater cardiometabolic risk burden.

**Table 1:** Baseline Characteristics by Stroke Status.

Characteristic	Non-Stroke (n = 5,962)	Stroke (n = 307)	*P*-value
*Demographics*			
Male	1282(21.5%)	63(20.5%)	0.683
Age(years)	58.00(51.00–65.00)	63.00(57.00–69.00)	
Urban residence	973(16.3%)	65(21.2%)	0.026
Married	5262(88.3%)	251(81.8%)	< 0.001
*Education Level*			
Illiterate	457(7.7%)	25(8.1%)	0.947
Primary school	573(9.6%)	30(9.8%)	
Secondary or higher	4932(82.7%)	252(82.1%)	
*Lifestyle Factors*			
Current smoker	579(9.7%)	26(8.5%)	0.472
Alcohol consumption	1489(25.0%)	60(19.5%)	0.031
Sleep(7–8 h)	1008(16.9%)	30(9.8%)	< 0.001
PA_2011_((MET-min/week)	0.00(0.00–1732.50)	0.00(0.00–1422.00)	0.8071
PA_2015_((MET-min/week)	1260.00(420.00–2100.00)	885.00(307.50–1462.50)	< 0.0001
PA_Δ_((MET-min/week)	750(0–1785)	525(− 102.20, 1236.9)	< 0.0001
*History of comorbidities*			
Hypertension	1494(25.1%)	134(43.6%)	< 0.001
Diabetes mellitus	285(4.8%)	44(14.3%)	< 0.001
Dyslipidemia	508(8.5%)	64(20.8%)	< 0.001
Renal disease	305(5.1%)	27(8.8%)	0.005
*Continuous Variables*			
LDL-C(mg/dL)	102.51(83.98–121.04)	108.49(90.54–126.45)	0.0042
CRP(mg/L)	1.30(0.45–2.15)	1.70(0.85–2.55)	< 0.0001
BMI(kg/m^2^)	24.14(21.74–26.53)	24.99(22.73–27.25)	0.0002
TyG-BMI	210.25(183.47–237.02)	221.94(194.50–249.39)	< 0.0001

Categorical variables were presented as n (%); *P*-values from chi-square tests; Continuous variables were presented as median (IQR); P-values from Wilcoxon rank-sum tests; Significant results (*P* < 0.05) were bolded; Dashes indicate within-group ranges not shown for continuous variables; PA, physical activity; MET-min/week, metabolic equivalent task minutes per week; TyG-BMI, triglyceride-glucose body mass index; LDL-C, low-density lipoprotein cholesterol; CRP, C-reactive protein; BMI,Body Mass Index;

### Single-pathway mediation analysis

We first performed single-pathway mediation analysis to LDL‑C, TyG‑BMI, and CRP separately as potential mediators of the association between physical activity change and stroke risk (Tables S1 – S2 and Table 2). In the crude model, all three biomarkers showed statisticall significantlly indirect effects, with mediated proportions of 2.44% for LDL‑C, 7.62% for TyG‑BMI, and 2.55% for CRP. After adjustment for age and sex, the mediation effect of TyG‑BMI remained evident, whereas the indirect effects through LDL‑C and CRP were attenuated mediation effect ofttenuated and no longer statistically significant.

**Table 2:** Single-pathway mediation effects of physical activity changes on stroke risk via LDL-C, TyG-BMI, and CRP.

Effect Type	LDL-C			TyG-BMI			CRP		
	Coefficient (β)	OR (95% CI)	Effect Proportion	Coefficient (β)	OR (95% CI)	Effect Proportion	Coefficient (β)	OR (95% CI)	Effect Proportion
Total Effect	–	–	–	− 0.141*	0.87(0.77,0.97)	100.00%	–	–	–
Direct Effect	− 0.126*	0.89(0.79,0.99)	–	− 0.126*	0.89(0.79,0.99)	89.47%	− 0.126*	0.89(0.79,0.99)	–
Indirect Effect	–	–	–	− 0.141*	0.985(0.976,0.995)	10.53%	–	–	–
*Path Decomposition*									
PA → Mediator (a)	− 0.022	–	–	− 0.067***	–	–	− 0.027*	–	–
Mediator → Stroke (b)	0.069	1.07(0.96,1.20)	–	0.223***	1.25(1.11,1.41)	–	0.005	1.01(0.89,1.13)	–

OR, Odds ratio represents the change in stroke incidence risk per unit variation in physical activity (PA);CI, confidence interval; Analyses were adjusted for age, gender, underlying diseases, educational level, hypertension, kidney disease, sleep status, and residential area. *P < 0.05, **P < 0.01, ***P < 0.001; Dashes indicate that the estimate was not applicable, not estimable.

In the fullly adjusted single-pathway model, TyG-BMI remained the only significant mediator. Greater physical activity changes was associated with lower TyG-BMI (β = −0.067), and higher TyG-BMI was associated with increased stroke risk (β = 0.223, OR = 1.25, 95%CI 1.11–1.41). The indirect effect through TyG-BMI accounted for 10.53% of the total association, while the direct effect remained significant (β = −0.126, OR = 0.89, 95%CI 0.79–0.99). By contrast, LDL-C and CRP did not showed significant mediation in the fully adjusted model. Physical activity change was not significantly associated with LDL-C (β = −0.022), and LDL-C was not significantly associated with stroke risk (β = 0.069, OR = 1.07, 95%CI 0.96–1.20). Although physical activity change was modestly associated with lowe CRP (β = -0.027), CRP was not associated with incident stroke(β = 0.005, OR = 1.01, 95%CI 0.89–1.13).

### Comprehensive mediation model

We next fitted a comprehensive mediation model including TyG-BMI, LDL-C, and CRP simultaneously as parallel mediators (Table 3). In the crude Model 1, TyG-BMI accounted for the largest mediated proportion (7.00%, β = −0.0165, OR = 0.98, 95%CI 0.97–0.99), followed by LDL-C (2.21%, β = −0.0052, OR = 0.99, 95%CI 0.99–1.00). The CRP pathway was not statistically significant.

**Table3:** Comprehensive Mediation Model for Physical Activity Reducing Stroke Risk.

Pathway	Model1			Model2			Model3		
	β Coefficient	OR (95% CI)	Effect Proportion	β Coefficient	OR (95% CI)	Effect Proportion	β Coefficient	OR (95% CI)	Effect Proportion
Total Effect	− 0.2356***	0.79(0.70,0.88)	100%	− 0.171**	0.843(0.749,0.937)	100%	− 0.141*	0.869(0.775,0.974)	100%
Direct Effect	− 0.2141***	0.81(0.72,0.90)	90.88%	− 0.144*	0.866(0.775,0.967)	84.3%	− 0.126*	0.881(0.787,0.987)	89.36%
*Indirect Effects*									
∟ TyG-BMI	− 0.0165**	0.98(0.97,0.99)	7.00%	− 0.027***	0.974(0.962,0.986)	15.7%	− 0.015**	0.985(0.976,0.995)	10.64%
∟ LDL	− 0.0052*	0.99(0.99,1.00)	2.21%	0.002	1.002(0.999,1.005)	–			
∟ CRP	− 0.0031	1.00(0.99,1.01)	1.32%	≈0	≈1.000	–	–	–	–
Total Indirect	− 0.0248***	0.98(0.96,0.99)	10.53%	− 0.025***	0.975(0.964,0.987)	15.7%	− 0.015**	0.985(0.976,0.995)	–

OR, Odds Ratio (OR) reflects the impact of per unit change in Physical Activity (PA) on the incidence risk of stroke; ;CI, confidence interval;Model 1 was adjusted for diabetes, educational level, hypertension, gender, kidney disease, sleep status, and residential area; Model 2 was adjusted for age, educational level, hypertension, gender, kidney disease, sleep status, and residential area; Model 3 was adjusted for diabetes, educational level, age, gender, kidney disease, sleep status, and residential area; *p < 0.05, **p < 0.01, ***p < 0.001; Dashes indicate that the estimate was not applicable, not estimable.

In the fully adjusted comprehensive Model, only the TyG-BMI pathway remained statistically significant (β = −0.015; OR = 0.985, 95%CI 0.976–0.995), accounting for 10.64% of the total association. The direct effect accounted for 89.36% of the association between physical activity change and incident stroke. These findings suggest that the association between physical activity and lower stroke risk against stroke is predominantly direct, with TyG-BMI serving as the key mediator.

### Causal diagram and subgroup validation

**Fig. 1:**
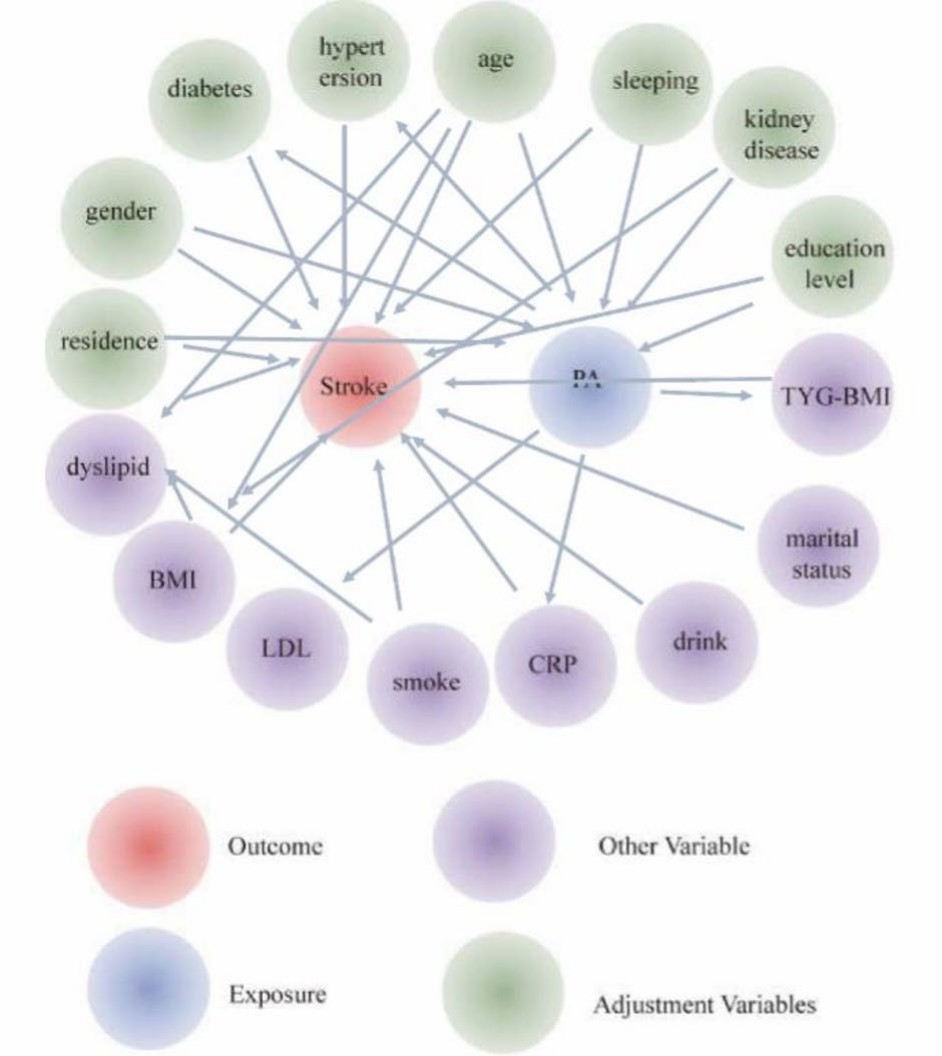
Directed acyclic graph (DAG) illustrating the assumed relationships among physical activity, incident stroke, candidate mediators, and potential confounders. Notes: The DAG guided the mediation analysis of the association between physical activity and stroke risk. Abbreviations: PA, physical activity; TyG-BMI, triglyceride-glucose body mass index; LDL-C, low-density lipoprotein cholesterol; CRP, C-reactive protein.

The directed Acyclic Graph (DAG) identified identified age, sex, residence, education level, sleep duration, hypertension, renal disease, and related health factors as potential confounders in the association between physical activity and stroke risk (Fig. 1). Based on the overall risk profile and mediation results, subgroup analyses were further conducted by age, hypertension status, and diabetes status (Table 4).

**Table 4:** Multidimensional Subgroup Analysis of Physical Activity on Stroke Risk.

Stratification	Subgroup	Direct Effect OR(95%CI)	P-interaction	TyG-BMI Mediation OR(95%CI)	P-interaction	LDL Mediation OR(95%CI)	P-interaction	CRP Mediation OR(95%CI)	P-interaction	ffect Decomposition(%)
*Age*^*1*^										
	≥ 60 years	0.96(0.93,0.99)**	0.004	0.996(0.993,0.999)**	0.009	1.002(0.998,1.006)	0.185	1.001(0.998,1.004)	0.412	83.7/14.5/1.2/0.6
	< 60 yrs	1.01(0.99,1.03)	–	1.000(0.998,1.002)	–	0.999(0.997,1.001)	–	0.999(0.997,1.001)	–	–
Hypertension^2^										89.2/9.3/1.1/0.4
	Yes	0.94(0.91,0.97)***	< 0.001	0.995(0.992,0.998)**	0.003	1.001(0.998,1.004)	0.214	1.000(0.997,1.003)	0.326	–
	No	1.02(0.99,1.05)	–	1.001(0.998,1.004)	–	0.998(0.995,1.001)	–	1.002(0.999,1.005)	–	88.5/10.2/1.1/0.2
*Diabetes*^*3*^										
	Yes	1.01(0.96,1.06)	0.032	1.00(0.99,1.01)	0.937	1.00(0.99,1.01)	0.652	1.00(0.99,1.01)	0.887	–
	No	0.99(0.98,1.00)*	–	0.999(0.998,1.000)**	–	1.00	–	–	–	–

OR, Odds Ratio (OR) reflects the impact of per unit change in Physical Activity (PA) on the incidence risk of stroke; ;CI, confidence interval;Model 1 was adjusted for diabetes, educational level, hypertension, gender, kidney disease, sleep status, and residential area; Model 2 was adjusted for age, educational level, hypertension, gender, kidney disease, sleep status, and residential area; Model 3 was adjusted for diabetes, educational level, age, gender, kidney disease, sleep status, and residential area; *p < 0.05, **p < 0.01, ***p < 0.001; Dashes indicate that the estimate was not applicable, not estimable.

Among participants aged ≥ 60 years, physical activity change was significantly associated with lower stroke risk through both the direct pathway(OR = 0.96, 95% CI 0.93–0.99) and the TyG-BMI-mediated pathway(OR = 0.996, 95% CI 0.993–0.999). The corresponding effect decomposition was 83.7% for the direct pathway and 14.5% for the TyG-BMI-mediated pathway. No significant mediation pathway was observed among participants aged < 60 years. In participants with hypertensive, the direct association between physical activity change and stroke risk was stronger (OR = 0.94, 95% CI 0.91–0.97), and the TyG-BMI-mediated pathway was also significant (OR = 0.995, 95% CI 0.992–0.998; P for interaction = 0.003). These associations were not evident among participants without hypertension.

Among patients with diabetes, none of the mediation pathways reached statistical significance. However, the interaction test for the direct pathway was significant (*P* = 0.032), suggesting that diabetes status may modify the association between physical activity change and stroke risk. Across all subgroup analyses, the LDL-C and CRP pathways were not statistically significant, consistent with the findings from the fully adjusted mediation models.

## Discussion

This nationwide longitudinal study examined potential metabolic, lipid, and inflammatory pathways linking physical activity (PA) with incident stroke in a middle-aged and older Chinese population. In the crude comprehensive mediation model, TyG-BMI, LDL-C, and CRP accounted for 7.00%, 2.21%, and 1.32% of the PA-stroke association, respectively. Query ID=“Q1” Text="Reference [41] is given in list but not cited in text. Please cite in text or delete from list." After full adjustment, however, only the TyG-BMI pathway remained statistically significant, mediating 10.64% of the association, whereas the LDL-C and CRP pathways were attenuated and no longer significant. These findings suggest that insulin resistance and adiposity-related metabolic dysfunction, as reflected by TyG-BMI, may represent the most robust measurable pathway among the three candidate mediators evaluated in this cohort. This metabolic mediation was stronger in participants aged ≥ 60 years and those with hypertension, but weaker in individuals with diabetes, suggesting heterogeneous protective pathways across subgroups. PA also demonstrated a significant direct protective effect, indicating additional uncharacterized biological pathways may be involved.

Our study are consistent with previous epidemiological evidence showing that higher PA is associated with lower stroke risk^27–30^. The present study extends this evidence by comparing metabolic disorders, lipid, and inflammatory mediators within the same analytical framework. TyG-BMI integrates fasting glucose, triglycerides, and BMI, and therefore captures both insulin resistance and adiposity-related metabolic burden more comprehensively than single biomarkers^31,32^. This may explain why TyG-BMI remained significant after multivariable adjustment, whereas LDL-C and CRP did not. From a biological perspective, regular PA may improve skeletal muscle glucose uptake, enhance insulin sensitivity, and reduce adiposity-related metabolic stress, which are closely related to lower TyG-BMI and may contribute to reduced cerebrovascular risk^33,34^.

The attenuation of LDL-C and CRP after adjustment deserves careful interpretation. LDL-C and CRP showed weak mediation in the crude model, but their effects were no longer significant after accounting for demographic characteristics, comorbidities, and other covariates. This pattern may reflect overlap among lipid, inflammatory, and metabolic pathways, as well as confounding by age, adiposity, chronic disease status, and medication use^35,36^. In addition, LDL-C and CRP were each measured at a single time point and may have been influenced by lipid-lowering therapy, the evolving acute-phase inflammatory response, and heterogeneity in stroke etiology^37^. Therefore, our results do not exclude roles for lipid metabolism or inflammation in stroke pathogenesis, but they suggest that these two single biomarkers did not independently explain the PA-stroke association in the fully adjusted mediation model.

Although the direct effect accounted for most of the PA-stroke association, this should not be interpreted as evidence for an unexplained novel biological mechanism. In mediation analysis, the direct effect represents the component of the exposure-outcome association not captured by the selected mediators^20^. Several plausible cardiovascular pathways were not assessed as potential mediators, including blood pressure regulation, arterial stiffness, endothelial dysfunction, autonomic imbalance, prothrombotic activity, sleep health, psychosocial factors, and additional metabolic or inflammatory markers^38–40^. Regular PA may increases blood flow shear stress, promotes endothelial nitric oxide synthase activation^41^ improve nitric oxide (NO)bioavailability^42^, reduce oxidative stress^43^, and delay vascular remodeling or carotid intima-media thickness progression^44^.

Subgroup analysis suggested that the TyG-BMI-mediated pathway was more evident among adults aged >  = 60 years and participants with hypertension, but not among those with diabetes. This pattern is biologically plausible because aging^45^ and hypertension^46^ are accompanied by cumulative metabolic dysfunction, vascular stiffness, and endothelial injury^47^, which may make metabolic improvement more relevant to cerebrovascular protection. In contrast, among participants with diabetes, long-standing metabolic impairment, vascular calcification, or endothelial dysfunction may weaken the observable association between PA, TyG-BMI, and stroke risk^48,49^.These subgroup findings should be interpreted cautiously, but they suggest that metabolic status and comorbidity burden may modify the pathways linking PA with stroke risk.

The clinical implications of these findings should be framed conservatively. This observational mediation analysis does not demonstrate that screening for or intervening on TyG-BMI, LDL-C, or CRP will improve stroke outcomes beyond PA promotion itself. Instead, TyG-BMI may be considered a useful candidate marker for future mechanistic and intervention studies evaluating how PA-related metabolic improvement relates to stroke prevention. At the public health level, the findings support continued efforts to promote regular PA among middle-aged and older adults, particularly those with hypertension or metabolic risk. However, biomarker-guided prevention strategies require confirmation in prospective studies with repeated measurements and intervention designs.

A key strength of this study was the integration of multiple mediating pathways, enabling a more comprehensive assessment of how physical activity(PA) may protect against stroke. To our knowledge, this is among the first nationally representative longitudinal analyses based on CHARLS to jointly assess TyG-BMI, LDL-C, and CRP as parallel mediators underlying the relationship between changes in physical activity and incident stroke. Our findings identify metabolic improvement and provided evidence for targeted exercise strategies for high-risk individuals with metabolic abnormalities. Nevertheless, residual confounding from diet and medication use cannot be excluded. Detailed information on medication use was not consistently available for all participants in the analytic sample, particularly for lipid-lowering agents such as statins and anti-inflammatory medications such as nonsteroidal anti-inflammatory drugs (NSAIDs). Consequently, pharmacological attenuation of the LDL-C and CRP pathways, as well as residual confounding, cannot be excluded. PA was self-reported, limiting causal inference and increasing susceptibility to measurement error. Moreover, converting IPAQ duration categories to midpoint values may have underestimated PA exposure among highly active participants. The open-ended highest category (≥ 4 h) was assigned a fixed value of 240 min. This approach could have introduced non-differential misclassification, potentially biasing the PA-stroke association and corresponding mediation effects towards the null. In addition, the triglyceride-glucose (TyG) index reflects a static metabolic state and may not capture dynamic metabolic variation. Finally, CHARLS did not consistently distinguish ischemic stroke from hemorrhagic stroke. Because LDL-C may have different associations with ischemic stroke and intracerebral hemorrhage, pooling these outcomes may have obscured subtype-specific lipid-mediated effects^8^. Future research could use continuous glucose monitoring to characterize dynamic metabolic changes and integrate metabolomics with vascular imaging in prospective studies. TyG-BMI-stratified intervention trials could further test the clinical relevance of the observed associations.

## Conclusion

In this longitudinal CHARLS analysis, PA was associated with lower incident stroke risk. In the fully adjusted comprehensive mediation model, TyG-BMI, but not LDL-C or CRP, remained a significant potential mediator, accounting for 10.64% of the association. These findings support TyG-BMI as a candidate metabolic pathway linking PA and stroke risk and should motivate future mechanistic and intervention studies.

## Supplementary Information

Below is the link to the electronic supplementary material.

Supplementary Material 1

## Data Availability

The data were collected from the 2011 and 2015 waves of the CHARLS survey, publicly available on the CHARLS website (https://charls.pku.edu.cn/).

## Abbreviations

TyG-BMI: Triglyceride-glucose index combined with body mass index
LDL-C: Low-density lipoprotein cholesterol
CRP: C-reactive protein
PA: Physical activity
CHARLS: China health and retirement longitudinal study
IPAQ: International physical; activity questionnaire
METs: Metabolic equivalents
GSEM: Generalized structural equation model
OR: Odds ratio
CI: Confidence interval
DAG: Directed acyclic graph
VPA: Vigorous physical activity
MPA: Moderate physical activity
LPA: Light physical activity
PAV: Physical activity volume
PI: Physical inactivity
IMT: Intima-media thickness
FMD: Flow-mediated dilation
AT: Aerobic training
RT: Resistance training
CGM: Continuous glucose monitoring
IR: Insulin resistance
eNOS: Endothelial nitric oxide synthase
NO: Nitric oxide
CKB: China kadoorie biobank
TyG: Triglyceride-glucose index
BMI: Body mass index
IQR: Interquartile range
NSAIDs: Nonsteroidal anti-inflammatory drugs
CDC: Center(s) for disease control and prevention

## References

[1] Hilkens, N. A., Casolla, B., Leung, T. W. & de Leeuw, F. E. Stroke. *Lancet* **403**(10446), 2820–2836 (2024). 38759664 10.1016/S0140-6736(24)00642-1

[2] Roth, G. A. et al. Global burden of cardiovascular diseases and risk factors, 1990–2019: Update from the GBD 2019 study. *J. Am. Coll. Cardiol.* **76**(25), 2982–3021 (2020). 33309175 10.1016/j.jacc.2020.11.010 PMC7755038

[3] Fan, M. et al. Association between active commuting and incident cardiovascular diseases in Chinese: A prospective cohort study. *J. Am. Heart Assoc.* **8**(20), e012556 (2019). 31576770 10.1161/JAHA.119.012556 PMC6818036

[4] Reddin, C. et al. Influence of age on the association of vascular risk factors with acute stroke (INTERSTROKE): A case-control study. *Lancet Healthy Longev.* **6**(6), 100709 (2025). 40617250 10.1016/j.lanhl.2025.100709

[5] Bull, F. C. et al. World health organization 2020 guidelines on physical activity and sedentary behaviour. *Br. J. Sports Med.* **54**(24), 1451–1462 (2020). 33239350 10.1136/bjsports-2020-102955 PMC7719906

[6] Lavie, C. J., Ozemek, C., Carbone, S., Katzmarzyk, P. T. & Blair, S. N. Sedentary behavior, exercise, and cardiovascular health. *Circ. Res.* **124**(5), 799–815 (2019). 30817262 10.1161/CIRCRESAHA.118.312669

[7] Lu, Z. et al. Insulin resistance estimated by estimated glucose disposal rate predicts outcomes in acute ischemic stroke patients. *Cardiovasc. Diabetol.* **22**(1), 225 (2023). 37633905 10.1186/s12933-023-01925-1 PMC10464388

[8] Sun, L. et al. Causal associations of blood lipids with risk of ischemic stroke and intracerebral hemorrhage in Chinese adults. *Nat. Med.* **25**(4), 569–574 (2019). 30858617 10.1038/s41591-019-0366-x PMC6795549

[9] Zhao, Q. et al. Cholesterol metabolic reprogramming mediates microglia-induced chronic neuroinflammation and hinders neurorestoration following stroke. *Nat. Metab.* **7**(10), 2099–2116 (2025). 40987840 10.1038/s42255-025-01379-7 PMC12552130

[10] Song, K. et al. Research status of triglyceride glucose-body mass index (TyG-BMI index). *Front. Cardiovasc. Med.* **12**, 1597112 (2025). 40756600 10.3389/fcvm.2025.1597112 PMC12315701

[11] Selvi, N. M. K. et al. Association of triglyceride-glucose index (TyG index) with HbA1c and insulin resistance in type 2 diabetes mellitus. *Maedica (Bucur)* **16**(3), 375–381 (2021). 34925590 10.26574/maedica.2021.16.3.375 PMC8643546

[12] Bian, K. et al. Association between triglyceride-glucose indices and ischemic stroke risk across different glucose metabolism statuses. *Diabetes Res. Clin. Pract.* **222**, 112064 (2025). 40010673 10.1016/j.diabres.2025.112064

[13] Jiang, L. et al. Assessment of six insulin resistance surrogate indexes for predicting stroke incidence in Chinese middle-aged and elderly populations with abnormal glucose metabolism: A nationwide prospective cohort study. *Cardiovasc. Diabetol.* **24**(1), 56 (2025). 39915878 10.1186/s12933-025-02618-7 PMC11804005

[14] Gaine, S. P., Quispe, R., Patel, J. & Michos, E. D. New strategies for lowering low density lipoprotein cholesterol for cardiovascular disease prevention. *Curr. Cardiovasc. Risk Rep.* **16**(9), 69–78 (2022). 36213094 10.1007/s12170-022-00694-y PMC9543364

[15] Lee, M. et al. Association between intensity of low-density lipoprotein cholesterol reduction with statin-based therapies and secondary stroke prevention: A meta-analysis of randomized clinical trials. *JAMA Neurol.* **79**(4), 349–358 (2022). 35188949 10.1001/jamaneurol.2021.5578 PMC8861901

[16] Ridker, P. M. A test in context: High-sensitivity C-reactive protein. *J. Am. Coll. Cardiol.* **67**(6), 712–723 (2016). 26868696 10.1016/j.jacc.2015.11.037

[17] Sbriscia, M. et al. Triglyceride glucose index predicts long-term mortality and major adverse cardiovascular events in patients with type 2 diabetes. *Cardiovasc. Diabetol.* **24**(1), 115 (2025). 40065340 10.1186/s12933-025-02671-2 PMC11895143

[18] Mann, S., Beedie, C. & Jimenez, A. Differential effects of aerobic exercise, resistance training and combined exercise modalities on cholesterol and the lipid profile: Review, synthesis and recommendations. *Sports Med.* **44**(2), 211–221 (2014). 24174305 10.1007/s40279-013-0110-5 PMC3906547

[19] Pedersen, B. K. & Saltin, B. Exercise as medicine: Evidence for prescribing exercise as therapy in 26 different chronic diseases. *Scand. J. Med. Sci. Sports* **25**(Suppl 3), 1–72 (2015). 26606383 10.1111/sms.12581

[20] VanderWeele, T. J. Mediation analysis: A practitioner’s guide. *Annu. Rev. Public Health* **37**, 17–32 (2016). 26653405 10.1146/annurev-publhealth-032315-021402

[21] Bai, W., Zhou, G., Jiang, H., Li, X. & Shao, J. Shared genetic architecture between stroke and blood lipids: A large-scale genome-wide cross-trait analysis. *Hum. Genomics* **19**(1), 75 (2025). 40605082 10.1186/s40246-025-00789-8 PMC12224841

[22] Yao, X. et al. Atherogenic index of plasma and high-sensitivity C-reactive protein: Combined effects on stroke risk in a middle-aged and elderly non-diabetic cohort. *Acta Neurol. Belg.*10.1007/s13760-026-03134-5 (2026). 42426492 10.1007/s13760-026-03134-5

[23] Craig, C. L. et al. International physical activity questionnaire: 12-country reliability and validity. *Med. Sci. Sports Exerc.* **35**(8), 1381–1395 (2003). 12900694 10.1249/01.MSS.0000078924.61453.FB

[24] Li, X. et al. Level of physical activity among middle-aged and older Chinese people: Evidence from the China health and retirement longitudinal study. *BMC Public Health* **20**(1), 1682 (2020). 33172439 10.1186/s12889-020-09671-9 PMC7653852

[25] Jetté, M., Sidney, K. & Blümchen, G. Metabolic equivalents (METS) in exercise testing, exercise prescription, and evaluation of functional capacity. *Clin. Cardiol.* **13**(8), 555–565 (1990). 2204507 10.1002/clc.4960130809

[26] Dou, J. et al. Association between triglyceride glucose-body mass and one-year all-cause mortality of patients with heart failure: A retrospective study utilizing the MIMIC-IV database. *Cardiovasc. Diabetol.* **22**(1), 309 (2023). 37940979 10.1186/s12933-023-02047-4 PMC10634170

[27] Joundi, R. A., Patten, S. B., Williams, J. V. A. & Smith, E. E. Association between excess leisure sedentary time and risk of stroke in young individuals. *Stroke* **52**(11), 3562–3568 (2021). 34407638 10.1161/STROKEAHA.121.034985

[28] Wei, J. et al. Metabolic factors mediate the causal effect of physical activity and sedentary behavior on stroke and its subtypes: Evidence from Mendelian randomization study. *Mol. Neurobiol.* **62**(8), 9892–9901 (2025). 40172820 10.1007/s12035-025-04881-x

[29] Jiang, C., Chen, T., Xiang, J. & Pang, Y. Association between physical activity levels and stroke risk among Chinese adults aged 45 and over based on CHARLS. *Sci. Rep.* **14**(1), 31739 (2024). 39738310 10.1038/s41598-024-81919-5 PMC11686039

[30] Shi, Y. et al. Association between physical activity patterns and the risk of stroke phenotypes: An accelerometer-based prospective cohort study from the UK Biobank. *BMC Public Health* **25**(1), 2593 (2025). 40739205 10.1186/s12889-025-23820-y PMC12309056

[31] Rao, X. et al. Triglyceride-glucose-body mass index and the incidence of cardiovascular diseases: A meta-analysis of cohort studies. *Cardiovasc. Diabetol.* **24**(1), 34 (2025). 39844258 10.1186/s12933-025-02584-0 PMC11756031

[32] Wang, B., Li, L., Tang, Y. & Ran, X. Joint association of triglyceride glucose index (TyG) and body roundness index (BRI) with stroke incidence: A national cohort study. *Cardiovasc. Diabetol.* **24**(1), 164 (2025). 40241070 10.1186/s12933-025-02724-6 PMC12004739

[33] Peifer-Weiß, L., Al-Hasani, H. & Chadt, A. AMPK and beyond: The signaling network controlling RabGAPs and contraction-mediated glucose uptake in skeletal muscle. *Int. J. Mol. Sci.*10.3390/ijms25031910 (2024). 38339185 10.3390/ijms25031910 PMC10855711

[34] Gao, Y. & Sun, W. The non-linear relationship between the metabolic score for insulin resistance and three-month outcomes in the individuals with acute ischemic stroke: A prospective Korean cohort study. *BMC Neurol.* **25**(1), 280 (2025). 40618038 10.1186/s12883-025-04299-x PMC12228382

[35] Aronson, D. et al. Obesity is the major determinant of elevated C-reactive protein in subjects with the metabolic syndrome. *Int. J. Obes. Relat. Metab. Disord.* **28**(5), 674–679 (2004). 14993913 10.1038/sj.ijo.0802609

[36] Rocha, V. Z. & Libby, P. Obesity, inflammation, and atherosclerosis. *Nat. Rev. Cardiol.* **6**(6), 399–409 (2009). 19399028 10.1038/nrcardio.2009.55

[37] Albert, M. A., Danielson, E., Rifai, N. & Ridker, P. M. Effect of statin therapy on C-reactive protein levels: The pravastatin inflammation/CRP evaluation (PRINCE): A randomized trial and cohort study. *JAMA* **286**(1), 64–70 (2001). 11434828 10.1001/jama.286.1.64

[38] Steptoe, A. & Kivimäki, M. Stress and cardiovascular disease. *Nat. Rev. Cardiol.* **9**(6), 360–370 (2012). 22473079 10.1038/nrcardio.2012.45

[39] Saz-Lara, A. et al. Association between sleep duration and sleep quality with arterial stiffness: A systematic review and meta-analysis. *Sleep Health* **8**(6), 663–670 (2022). 36055936 10.1016/j.sleh.2022.07.001

[40] Tanayapong, P. & Kuna, S. T. Sleep disordered breathing as a cause and consequence of stroke: A review of pathophysiological and clinical relationships. *Sleep Med. Rev.* **59**, 101499 (2021). 34020180 10.1016/j.smrv.2021.101499

[41] Hambrecht, R. et al. Regular physical activity improves endothelial function in patients with coronary artery disease by increasing phosphorylation of endothelial nitric oxide synthase. *Circulation* **107**(25), 3152–3158 (2003). 12810615 10.1161/01.CIR.0000074229.93804.5C

[42] Carlström, M., Weitzberg, E. & Lundberg, J. O. Nitric oxide signaling and regulation in the cardiovascular system: Recent advances. *Pharmacol. Rev.* **76**(6), 1038–1062 (2024). 38866562 10.1124/pharmrev.124.001060

[43] Iring, A. et al. Shear stress-induced endothelial adrenomedullin signaling regulates vascular tone and blood pressure. *J. Clin. Invest.* **129**(7), 2775–2791 (2019). 31205027 10.1172/JCI123825 PMC6597232

[44] Wang, Y. et al. Effect of exercise on carotid artery intima-media thickness in adults: A systematic review and meta-analysis. *J. Phys. Act. Health* **19**(12), 855–867 (2022). 36257606 10.1123/jpah.2022-0372

[45] Donato, A. J., Machin, D. R. & Lesniewski, L. A. Mechanisms of dysfunction in the aging vasculature and role in age-related disease. *Circ. Res.* **123**(7), 825–848 (2018). 30355078 10.1161/CIRCRESAHA.118.312563 PMC6207260

[46] Yannoutsos, A., Levy, B. I., Safar, M. E., Slama, G. & Blacher, J. Pathophysiology of hypertension: Interactions between macro and microvascular alterations through endothelial dysfunction. *J. Hypertens.* **32**(2), 216–224 (2014). 24270179 10.1097/HJH.0000000000000021

[47] Pasha, E. P., Birdsill, A. C., Oleson, S., Haley, A. P. & Tanaka, H. Impacts of metabolic syndrome scores on cerebrovascular conductance are mediated by arterial stiffening. *Am. J. Hypertens.* **31**(1), 72–79 (2017). 28992237 10.1093/ajh/hpx132 PMC5861594

[48] Yang, D. R., Wang, M. Y., Zhang, C. L. & Wang, Y. Endothelial dysfunction in vascular complications of diabetes: A comprehensive review of mechanisms and implications. *Front Endocrinol (Lausanne)* **15**, 1359255 (2024). 38645427 10.3389/fendo.2024.1359255 PMC11026568

[49] Way, K. L., Keating, S. E., Baker, M. K., Chuter, V. H. & Johnson, N. A. The effect of exercise on vascular function and stiffness in type 2 diabetes: A systematic review and meta-analysis. *Curr. Diabetes Rev.* **12**(4), 369–383 (2016). 26279493 10.2174/1573399811666150817124601

