# Supplementary material for: Mediation effect of TyG-BMI, LDL-C, and CRP on physical activity-stroke risk relationship
Source: Sci Rep. 2026 Jul 23;16:23352. doi: 10.1038/s41598-026-63670-1 (PMC13408081; doi:10.1038/s41598-026-63670-1)
Supplement: Supplementary file 1 — Supplementary Material 1 [file 41598_2026_63670_MOESM1_ESM.docx]

**Table S1. Mediation effects of physical activity on stroke risk via LDL-C, TyG-BMI, and CRP(crude model)**

| Effect Type | LDL-C | | | TyG-BMI | | | CRP | | |
| --- | --- | --- | --- | --- | --- | --- | --- | --- | --- |
|  | Coefficient (β) | OR (95% CI) | Effect Proportion | Coefficient (β) | OR (95% CI) | Effect Proportion | Coefficient (β) | OR (95% CI) | Effect Proportion |
| Total Effect | -0.2353*** | 0.79(0.71,0.88) | 100% | -0.2376*** | 0.79(0.70,0.88) | 100% | -0.2356*** | 0.79(0.71,0.88) | 100% |
| Direct Effect | -0.2296*** | 0.80(0.71,0.89) | 97.56% | -0.2195*** | 0.80(0.72,0.90) | 92.38% | -0.2296*** | 0.79(0.71,0.89) | 97.45% |
| Indirect Effect | -0.0057* | 0.99(0.99,1.00) | 2.44% | -0.0181*** | 0.98(0.97,0.99) | 7.62% | -0.0060* | 0.99(0.99,1.00) | 2.55% |
| Path Decomposition |  |  |  |  |  |  |  |  |  |
| PA → Mediator (a) | -0.0444*** | - | - | -0.0745*** | - | - | -0.0419*** | - | - |
| Mediator → Stroke (b) | 0.1293* | 1.14(1.02,1.28) | - | 0.223*** | 1.25(1.11,1.41) |  | 0.1437* | 1.15(1.03,1.29) | - |

Note: All PA -> mediator path coefficients are reported on the same standardized/model scale. PA was expressed as MET-min/week according to the IPAQ-derived PAV calculation; Odds Ratio (OR) reflects per unit change in physical activity (PA) on stroke incidence risk. The crude model included no covariates

**Table S2. Mediation effects of physical activity on stroke risk via LDL-C, TyG-BMI, and CRP (adjusted for age and gender)**

| Effect Type | LDL-C | | | TyG-BMI | | | CRP | | |
| --- | --- | --- | --- | --- | --- | --- | --- | --- | --- |
|  | Coefficient (β) | OR (95% CI) | Effect Proportion | Coefficient (β) | OR (95% CI) | Effect Proportion | Coefficient (β) | OR (95% CI) | Effect Proportion |
| Total Effect | - | - | - | -0.1707** | 0.843(0.749,0.937) | 100% | - |  | - |
| Direct Effect | -0.1691** | 0.844(0.751,0.950) | - | -0.1452* | 0.865(0.767,0.975) | 85.07% | -0.1684** | 0.845(0.751,0.951) | - |
| Indirect Effect | - | - | - | -0.0255*** | 0.975(0.964,0.987) | 14.93% | - |  | - |
| Path Decomposition |  |  |  |  |  |  |  |  |  |
| PA → Mediator (a) | -0.0256* | 0.957(0.934,0.980) |  | -0.0341*** | - | - | -0.0329** | - |  |
| Mediator → Stroke (b) | 0.0896 | 1.094(0.973,1.230) |  | 0.3089*** | 1.362(1.216,1.525) |  | 0.1093* | 1.115(0.994,1.251) |  |

Note: All PA -> mediator path coefficients are reported on the same standardized/model scale. PA was expressed as MET-min/week according to the IPAQ-derived PAV calculation; Odds Ratio (OR) reflects per unit change in physical activity (PA) on stroke incidence risk. Adjustments were made for age, gender. *P < 0.05, **P < 0.01, ***P < 0.001.
